# Multiomics analysis of adaptation to repeated DNA damage in prostate cancer cells

**DOI:** 10.1080/15592294.2023.2214047

**Published:** 2023-05-17

**Authors:** D. Challis, T. Lippis, R. Wilson, E. Wilkinson, J. Dickinson, A. Black, I. Azimi, A. Holloway, P. Taberlay, K. Brettingham-Moore

**Affiliations:** a Tasmanian School of Medicine, University of Tasmania, Hobart, Tasmania, Australia; b Central Science Laboratory, University of Tasmania, Hobart, Tasmania, Australia; c Menzies Institute of Medical Research, University of Tasmania, Hobart, Tasmania, Australia; d Medical Oncology, Royal Hobart Hospital, Hobart, Tasmania, Australia; e School of Pharmacy and Pharmacology, University of Tasmania, Hobart, Tasmania, Australia

**Keywords:** DNA damage, DNA methylation, transcriptome, Proteome, Prostate cancer

## Abstract

DNA damage is frequently utilized as the basis for cancer therapies; however, resistance to DNA damage remains one of the biggest challenges for successful treatment outcomes. Critically, the molecular drivers behind resistance are poorly understood. To address this question, we created an isogenic model of prostate cancer exhibiting more aggressive characteristics to better understand the molecular signatures associated with resistance and metastasis. 22Rv1 cells were repeatedly exposed to DNA damage daily for 6 weeks, similar to patient treatment regimes. Using Illumina Methylation EPIC arrays and RNA-seq, we compared DNA methylation and transcriptional profiles between the parental 22Rv1 cell line and the lineage exposed to prolonged DNA damage. Here we show that repeated DNA damage drives the molecular evolution of cancer cells to a more aggressive phenotype and identify molecular candidates behind this process. Total DNA methylation was increased while RNA-seq demonstrated these cells had dysregulated expression of genes involved in metabolism and the unfolded protein response (UPR) with Asparagine synthetase (ASNS) identified as central to this process. Despite the limited overlap between RNA-seq and DNA methylation, oxoglutarate dehydrogenase-like (OGDHL) was identified as altered in both data sets. Utilising a second approach we profiled the proteome in 22Rv1 cells following a single dose of radiotherapy. This analysis also highlighted the UPR in response to DNA damage. Together, these analyses identified dysregulation of metabolism and the UPR and identified ASNS and OGDHL as candidates for resistance to DNA damage. This work provides critical insight into molecular changes which underpin treatment resistance and metastasis.

## Introduction

Following cancer diagnosis, patient response to treatment and prognosis is uncertain. Therapeutic interventions, such as chemotherapy or radiotherapy, frequently rely on repeated DNA damage to induce cancer cell death. Unfortunately, in cases of treatment resistance, this can result in repeated DNA repair which has the potential to alter the molecular profile of the surviving cell population. Molecular evolution during the treatment period therefore has the potential to contribute to more aggressive disease in the longer term. Supporting this concept, increased treatment resistance and migration of prostate cancer cells has been demonstrated in cell populations surviving recurrent damage [1,2].

Despite intensive research efforts over the past decades to identify predictive markers of treatment response and prognosis in cancer, few have reached clinical utility. While patient biopsies can be informative, often the genetic heterogeneity between patients and within tumours creates ‘noise’ and hinders successful identification of markers. The question remains, how do we resolve these challenges to identify the changes responsible for treatment response and prognosis? To understand how cells survive and potentially evolve during treatment it is necessary to determine how repeated DNA damage influences their molecular profile. Analysing the changes in cell behaviour and gene expression over an extended period of DNA damage will provide insight into the key factors driving cell survival.

We have previously reported a large transcriptional response to a *single* dose of irradiation along with stable epigenetic changes [3,4]. This raises the question, how does the molecular profile evolve in response to cycles of repeated DNA damage and repair? This is particularly relevant as most cancer patients receive recurrent fractionated treatment. We have generated an isogenic model of 22Rv1 prostate cancer cells to identify potential drivers of treatment resistance and metastasis to inform selection of predictive biomarkers. The radiomimetic and glycopeptide, phleomycin, was used to induce double-strand breaks (DSBs) in DNA [3,5] on a daily basis for a period of six weeks. The aim of this analysis was to provide a comprehensive insight into molecular changes that occur in response to DNA damage. The impact of repeated DNA damage and repair on cell survival and migratory potential was assessed along with the evolution of the transcriptional and epigenetic profile of the cells using the Illumina NovaSeq 6000 and EPIC array platforms. The clinical relevance of this data was further supported by proteomics analysis of a single 2 gray (Gy) dose of radiotherapy, with the dysregulated pathways identified supporting the RNA-seq data. Ultimately, this will aid in understanding how cells surviving DNA damaging treatments undergo molecular evolution which may contribute to future treatment resistance and metastasis.

## Results

### Prostate cancer cells exposed to repeated DNA damage have increased survival and invasive potential.

**Figure 1.**
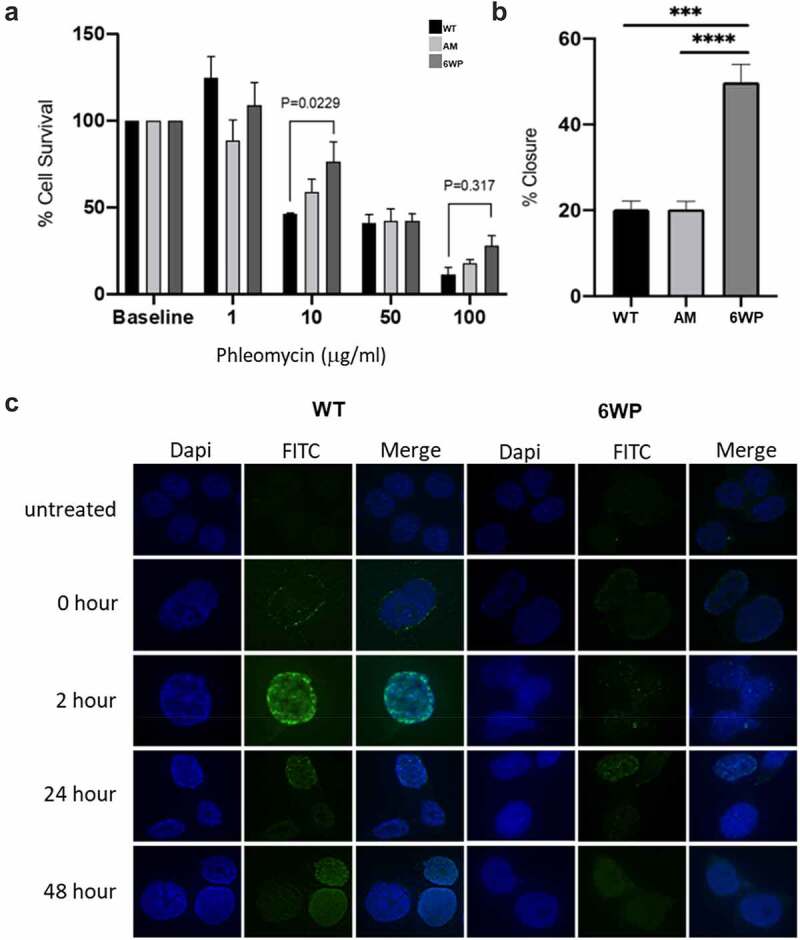
Repeated DNA damage increases cell survival and invasive potential. A) 22Rv1 cells were treated daily with 1 μg/ml of phleomycin for 6 weeks (6WP) or left untreated (WT, AM). A total of 1000 cells were treated with the indicated concentrations of phleomycin for 1 hour. Colonies were stained following 12 d of growth and percentage survival was calculated. The mean and SEM of three biological replicates is shown. Significance was determined by two-way ANOVA and Sidak’s post-test. *P < 0.05. B) WT, AM and 6WP cells were grown on chamber slides, scratched, and imaged at 0 and 48 hours. Results represent the percentage of the scratch width covered by migration at 48 hours relative to 0 hours. The mean and SEM of 3 biological and 3 technical replicates are shown. Significance was determined using a Student’s one-tailed t-test. *** *P* < 0.0001. C) Immunofluorescence staining for γH2A.X foci (FITC) in WT and 6WP cells following recovery from phleomycin-induced DNA damage at the indicated time points.

To establish an isogenic model of resistance to DNA damage, 22Rv1 prostate cancer cells were treated daily with 1 µg/ml of phleomycin for 6 weeks. In order to assess resistance of these cells to DNA damage, clonogenic survival was assessed following 6 weeks of treatment (6WP), as well as in parental/wild type (WT) and age matched (AM) control cells at the indicated doses (*n* = 3). Cell survival generally decreased with increasing phleomycin concentrations for all cell types, which demonstrated phleomycin was functioning as expected (Figure 1a). Phleomycin function was also supported by immunofluorescence data illustrating increased γH2A.X foci, and therefore double-strand breaks (DSBs), in response to phleomycin treatment (Figure S1). Following treatment at 10 µg/ml, 6WP cells displayed 76% survival compared to 59% survival for AM controls and 46% for WT controls (Figure 1a). The difference in survival between WT controls and 6WP cells was statistically significant (*P* = 0.0229). Phleomycin treatment at 50 µg/ml further decreased cell survival, although approximately 40% survival was observed for all cell types (Figure 1a). Following drug treatment at 100 µg/ml, 6WP cells showed 28% survival compared to 18% and 11% survival for AM and WT controls respectively (Figure 1a). Here the difference in survival between WT and 6WP cells was not statistically significant (*P* = 0.317). Overall, these data indicate that 6WP cells can generate more viable colonies than control cells when treated with 10 µg/ml of phleomycin, indicating resistance to DNA damage.

Next, the metastatic potential of these cells was assessed using a scratch assay. Cells were grown to confluency on chamber slides and monolayers were scratched with a pipette tip. Images captured at 0 and 48 hours were then compared to determine the percentage closure (n = 3, Figure 1b). At 48 hours 6WP cells showed 50% closure compared to 20% closure for both AM and WT controls (Figure 1b). These differences were highly significant (P = 0.0005 and *P*=<0.0001) suggesting increased migratory potential of cells surviving repeated DNA damage. Further investigation of response to phleomycin-induced DNA damage in the WT vs 6WP cells, using γH2A.X immunofluorescence, indicated that fewer DNA damage foci were evident over a 48 hour period in the 6W treated cells (Figure 1c).

### Prostate cancer cells surviving repeated DNA damage have increased DNA methylation

We have previously demonstrated that a single dose of radiotherapy alters the transcriptome and stably alters DNA methylation in prostate cancer cells [3,4]. We therefore became interested in how repeated exposure to and recovery from DNA damage could influence the DNA methylation profile of cells. 22Rv1s were exposed to daily DNA damage using the radiomimetic, phleomycin, over a 6-week period and the molecular evolution of cells during treatment was profiled using DNA methylation analysis (Illumina EPIC arrays, *n* = 2). To estimate the methylation status of a CpG, methylated and unmethylated probes are used and the intensity of each of the probes is measured [6]. The beta-value represents the ratio of the methylated probe intensity and the total signal intensity. Beta-values range from 0 to 1, with 0 representing 0% methylation and 1 representing 100% methylation [6].

**Figure 2.**
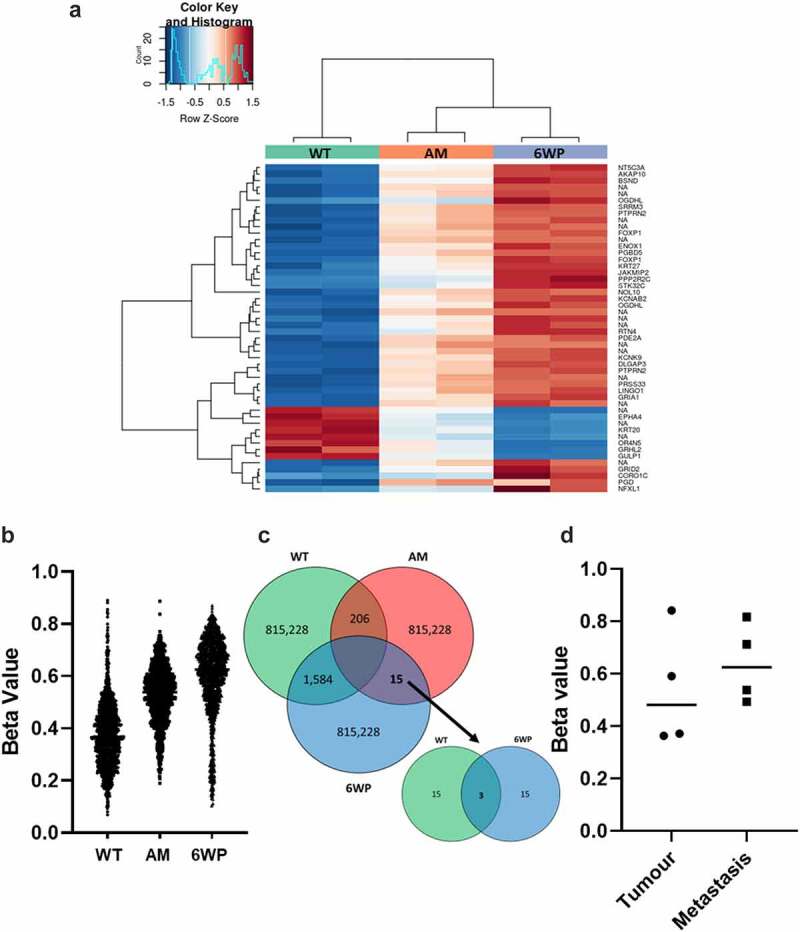
Repeated DNA damage in 22Rv1 cells increases DNA methylation. 22Rv1 cells were treated daily with 1 μg/ml of phleomycin for 6 weeks (6WP) or left untreated (WT, AM). DNA was extracted and DNA methylation was profiled by the Australian Genome Research Facility (AGRF) using the Illumina EPIC 850k array. A) Heatmap of top 50 most differentially methylated CpGs. Red indicates high levels of methylation and blue low methylation relative to the mean for a given CpG. CpGs not associated with a known gene are labelled ‘NA.’ B) Plot of over 1500 CpGs in 22Rv1 prostate cancer cells that displayed a beta change >0.25 after 6 weeks of daily DNA damage with a radiomimetic (1= fully methylated; 0= unmethylated). C) intersection of probes across the treatment groups. D) Beta values for OGDHL gene body methylation in four matched primary prostate and metastatic tumours (probe cg08846770 from EPIC array). Data was analysed via students t-test (ns).

Heatmaps generated depict methylation levels as Z-Scores, which represent the number of standard deviations from the mean level of methylation (Figure 2a), AM data is included for comparison. The gene names for the top 50 differentially methylated genes are included in Table S1. The top 50 DNA methylation changes which occur in cell culture in the absence of DNA damage (WT vs AM) are displayed in Figure S2.

The samples were not overly distinct in their global DNA methylation patterns. No significantly differentially methylated CpGs were identified using the corrected p-values. To interrogate the data further, probes with an uncorrected P-value <0.05 were identified. It is important to note that these are not considered statistically significant, however, they are useful in exploratory analysis. For probes with an uncorrected P-value <0.05, beta-values were averaged across replicates and filtered for a > 0.25 beta-value difference between treatment groups. When comparing WT controls and 6WP cells 1584 differentially methylated CpGs were identified, and these are plotted in Figure 2b along with AM for comparison. Between WT controls and AM controls 206 differentially methylated CpGs were identified. Finally, across the entire array, between AM controls and 6W phleomycin treated cells only 15 CpGs were differentially methylated using a cut-off of 0.25. Of these, only 3 CpGs were also differentially methylated between WT and 6W treated cells (Figure 2c), and only 1 CpG was associated with a known gene. This gene was Oxoglutarate Dehydrogenase-Like (*OGDHL*). To determine whether the increased *OGDHL* gene body methylation may be associated with more aggressive disease, matched primary prostate tumours and bone metastases (pairs from the same patients), previously profiled by Wilkinson et al. [7] using the Illumina MethylationEPIC beadchip, were interrogated for the two OGDHL probes cg08846770 and cg00898123 identified in our analysis. Unfortunately probe cg00898123 could not be investigated as it had been removed from analysis due to poor quality. A slight increase in DNA methylation at cg08846770was observed in metastatic disease, however this was not significant at *n* = 4 (Figure 2d).

### Prostate cancer cells surviving repeated DNA damage dysregulate expression of genes involved in the unfolded protein response

Next, global transcriptional changes were profiled in our model of repeated DNA damage. RNA was extracted from 22Rv1 cells treated daily for 6 weeks with 1 μg/ml of phleomycin (6WP) and from WT and AM control cells (*n* = 2). Following QC and library preparation, RNA-sequencing was conducted using the Illumina NovaSeq 6000 platform.

**Figure 3.**
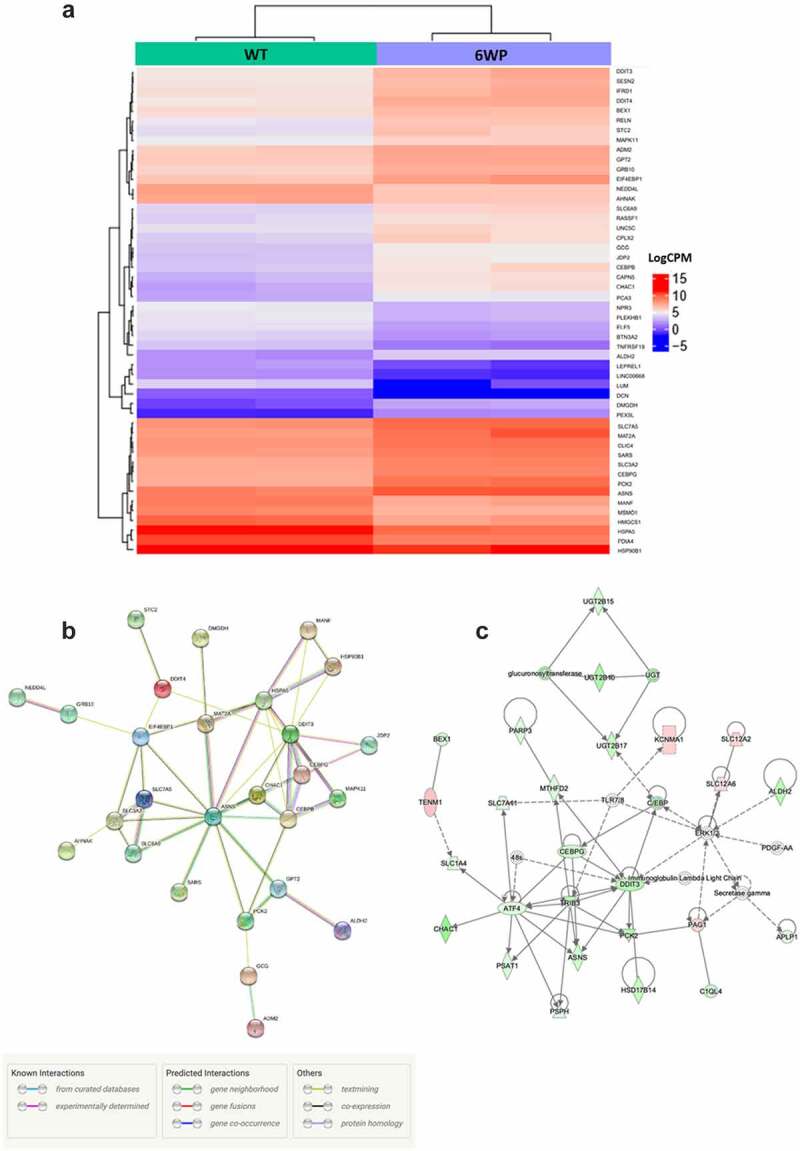
Repeated DNA damage alters transcriptional profile of 22Rv1 cells. 22Rv1 cells were treated daily with 1 μg/ml of phleomycin for 6 weeks (6WP) or left untreated (WT, AM). RNA was extracted and sequenced on the Illumina NovaSeq 6000 platform by the AGRF to generate 100 bp single end reads. A) Heatmap of log count per million (logCPM) values for the top 50 differentially expressed genes based on adjusted p-values. High gene expression is indicated by red and low expression is indicated by blue. Genes and experimental samples with similar expression patterns are clustered together. B) STRING pathway analysis showing interactions between the top 50 genes. C) The most significant IPA network for differential gene expression. Direct (solid line) and indirect (dashed line) connections between the top 500 differentially expressed genes were formed. Green nodes indicate upregulation and red nodes indicate downregulation of the corresponding gene.

To visualize differential gene expression in response to recurrent DNA damage the WT and 6WP treated samples were compared. The heatmap for the top 50 most differentially expressed genes demonstrates that expression levels for each gene were mostly similar between replicates and gene expression changes are subtle (Figure 3a). String pathway analysis using the top 50 genes identified Asparagine synthetase *(ASNS)* as key in this response (Figure 3b). Network relationships between the top 500 differentially expressed genes (WT vs 6WP) were also investigated using Ingenuity Pathway Analysis. The most significant gene network was the unfolded protein response pathway (Figure 3c). Interrogating the RNA-seq data further, *OGDHL* which was identified as potential candidate for surviving chronic DNA damage in our previous DNA methylation analysis, was found to be significantly upregulated in 6WP cells compared to WT controls (*P* = 0.0003). This correlates with the increased methylation at the CpG site within the gene body reported in Figure 2.

### Proteomics profiling reveals increases in cell adhesion and the unfolded protein response in prostate cancer cells exposed to a single dose of radiotherapy

**Figure 4.**
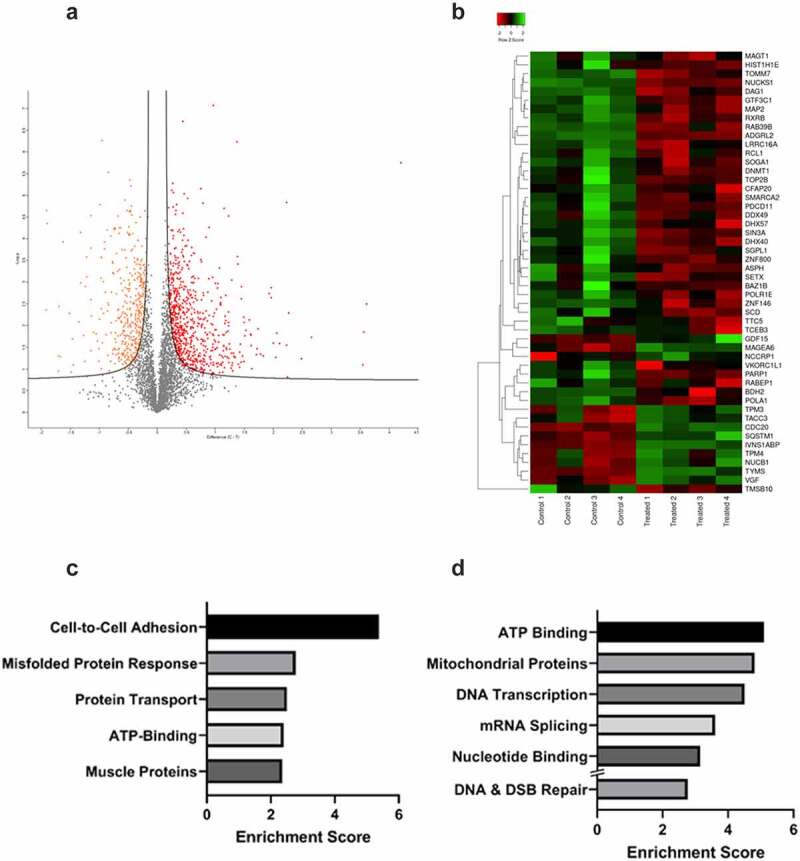
Proteomics analysis of 22Rv1 prostate cancer cells following a single 2 gray dose of radiotherapy. 22Rv1 cells were either left untreated or irradiated with a 2 Gy dose before extracting protein and analysing via HPLC/MS (n = 4). A) Volcano plot representing the fold change of proteins identified by HPLC-MS following irradiation of 22Rv1 cells. The statistical significance of the fold change was assessed using the Student’s t-test. Fold change was plotted against the -log10 *P* value using Perseus 1.6.15.0. Each point represents a single protein, a red point signifies the protein had significantly increased abundance, while an orange point signifies the inverse. FDR = 0.05, s0 = 0.1. C-T =control-treated. B) Heatmap showing fold change in protein abundance and their relatedness. The log change in protein abundance was calculated and Student t-tests were performed to determine proteins with a significant fold change. The fold change was normalized and plotted on the heatmap using www.heatmapper.ca. The abundance of each protein is indicated by the colour, with low abundance represented in green, and higher abundance in red. C & D) Functional clustering of proteins in irradiated 22Rv1 samples as compared to control. Proteins with a statistically significant fold change were divided into increased or decreased abundance groups. These proteins were submitted separately to DAVID and functional annotation clustering analysis was performed. For each respective set of proteins, the top five clusters by enrichment score are shown above. C) Shows the top five functional clusters by enrichment score as identified in the increased protein abundance group while D) shows the top five functional clusters by enrichment score as identified in the decreased protein abundance group. Additionally, DNA & DSB Repair functional cluster (ranked 7^th^) was also included as a cluster of interest.

The impact of DNA damage induced by radiotherapy was then assessed. 22Rv1 cells were exposed to a single 2 Gy dose of irradiation. Whole-cell protein was extracted from untreated and irradiated cells (*n* = 4) 24 hours later and analysed via High Performance Liquid Chromatography-Mass Spectrometry (HPLC-MS). Proteins were identified using the Uniprot database. 4053 proteins were identified and quantified in each sample with less than 0.02% of the proteins present in only one sample. Following protein identification, the data was imported into Perseus v1.6.15.0 (https://maxquant.net/perseus/) for further analysis. First, proteins with statistically significant changes in abundance between the treated and control groups were determined using multiple comparison t-tests. The FDR was set at 0.05 and the s0 at 0.1 to exclude proteins with very small differences between means. Of the 4053 proteins identified, 1141 proteins had significant changes in abundance following irradiation, with 414 proteins showing a significant decrease in abundance and the remaining 727 proteins showing increased abundance. This can be visualized in the volcano plot which shows the degree of statistical significance and fold change for every protein identified (Figure 4a). A heatmap of the top 50 proteins was also generated to visualize changes and patterns in protein abundance (Figure 4b).

**Figure 5.**
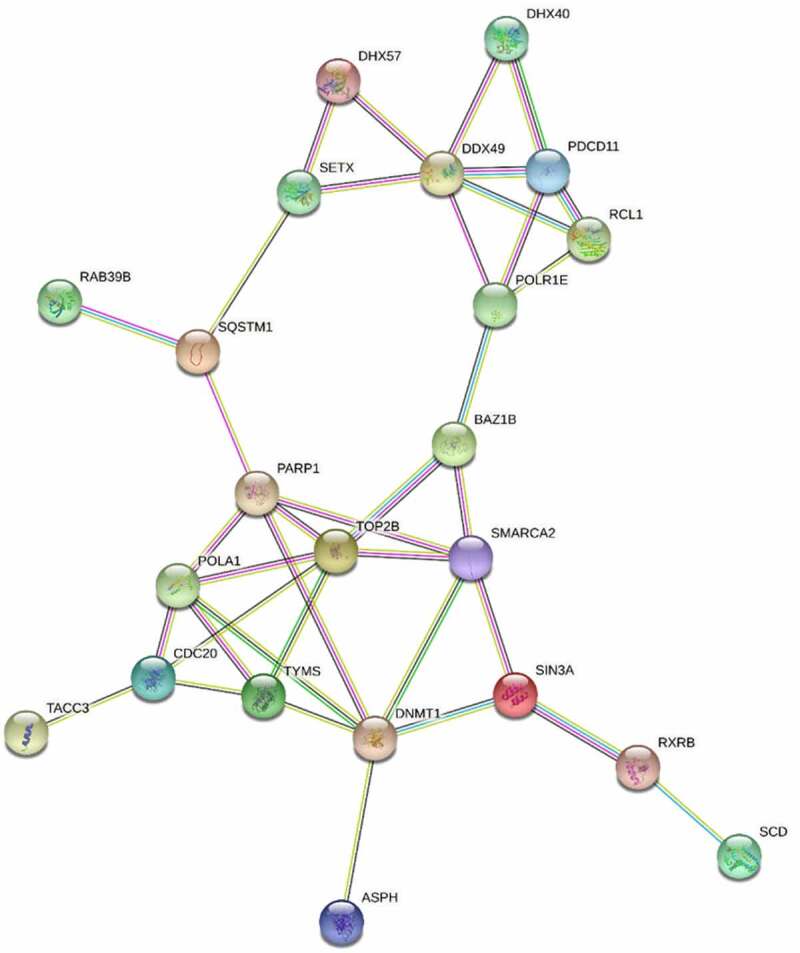
STRING analysis of the top 50 proteins with altered abundance following radiotherapy. Whole cell protein extracts prepared from untreated or irradiated 22Rv1 prostate cancer cells (24 hours following 2 Gy dose). Proteins were analysed via HPLC-MS and the 50 proteins with greatest change in abundance were then analysed via string-db.Org.

Functional annotation clustering was then used for bioinformatic analysis of the proteins with increased (Figure 4c) versus decreased abundance (Figure 4d). This analysis identified cell adhesion, misfolded protein response, protein transport, ATP binding, and muscle proteins as increased. Functional clusters for proteins with decreased abundance included ATP binding, mitochondrial proteins, transcription, mRNA splicing and nucleotide binding. While only the top 5 functional clusters were selected for analysis, one further cluster was included in the decreased abundance group. The DNA and double stranded break repair functional cluster was the 7th cluster by enrichment score in the decreased abundance group and was included due to its significance to this study. STRING analysis of the top 50 proteins revealed key interactions in this response including TOP2B, PARP1, POLA1 and epigenetic proteins DNMT1 and SMARCA2 (Figure 5).

To assess the similarities between the single-dose RT and adaptive response to DNA damage, the proteomics data was integrated with the DNA methylation and RNA-seq data sets. First, the two genes of interest from the DNA methylation and RNA-seq analysis, *OGDHL* and *ASNS*, were analysed. Comparison of this data revealed that while OGDHL was not identified as differentially expressed as determined by the proteomic analysis, ASNS was identified as being significantly increased in response to radiotherapy (1.3-fold, q value = 0.048). Next, overlap between the top 500 genes and proteins in terms of differential abundance from the RNA-seq and proteomics data was assessed, with 18 transcripts/proteins found to be shared (Table 1).

**Table 1.** Shared transcripts/proteins between RNA-seq and proteomics data sets.

DEAD-box helicase 49 (DDX49)
Glycine-N-acyltransferase like 1 (GLYATL1)
Membrane metalloendopeptidase (MME)
3-hydroxy-3-methylglutaryl-CoA synthase 1 (HMGCS1)
Apoptotic peptidase activating factor 1 (APAF1)
Growth differentiation factor 15 (GDF15)
RAB39B, member RAS oncogene family (RAB39B)
Interferon regulatory factor 2 binding protein 2 (IRF2BP2)
SAM and HD domain containing deoxynucleoside triphosphate triphosphohydrolase 1 (SAMHD1)
Aldehyde dehydrogenase 1 family member L2 (ALDH1L2)
Senataxin (SETX)
VGF nerve growth factor inducible (VGF)
Protein kinase cAMP-dependent type II regulatory subunit beta (PRKAR2B)
Argonaute 2, RISC catalytic component (AGO2)
Methyltransferase like 7B (METTL7B)
Methyltransferase like 7A (METTL7A)
Vimentin (VIM)
Phospholipid transfer protein (PLTP)

### OGDHL and ASNS expression is decreased in response to a single dose of radiotherapy but increased in response to repeated DNA damage

**Figure 6.**
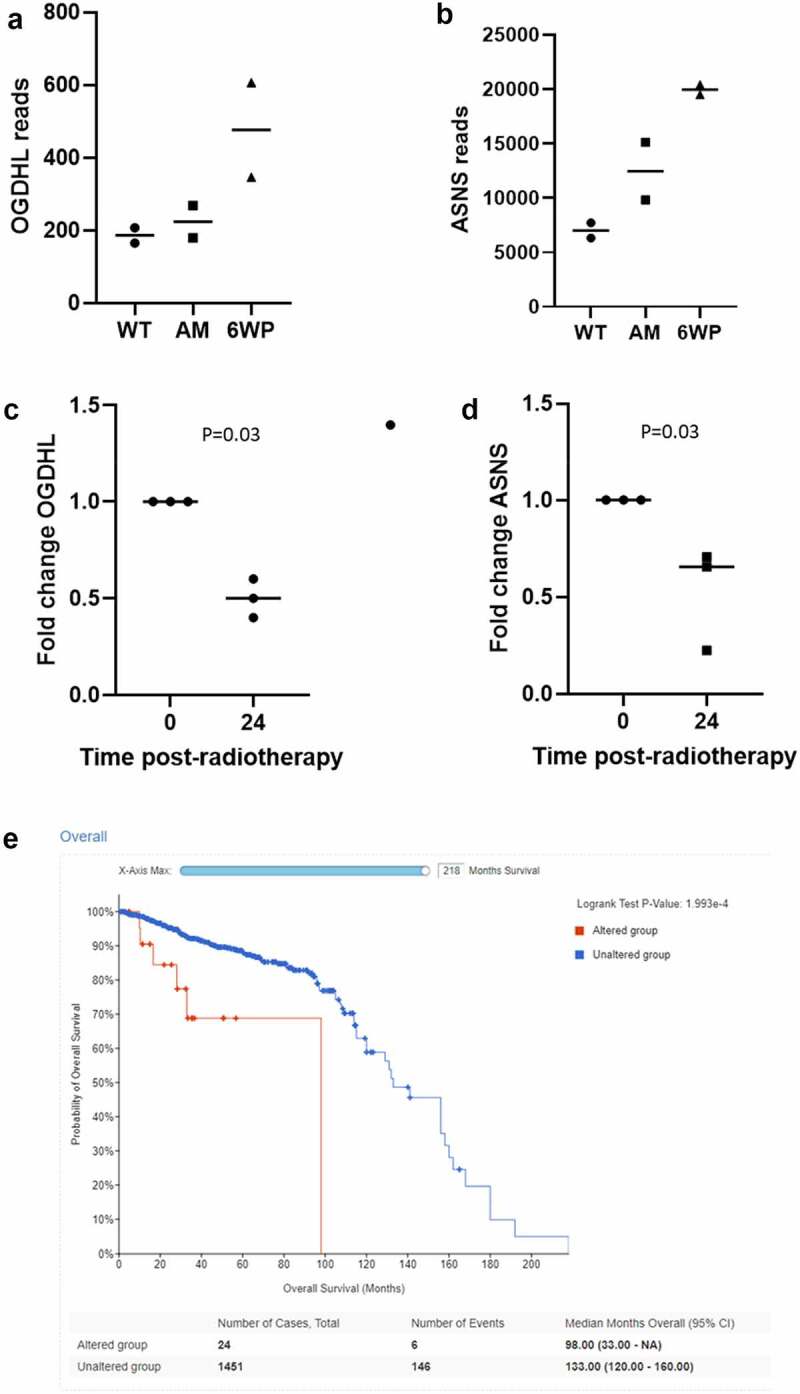
OGHDL and ASNS expression profiling following repeated DNA damage versus a single dose of radiotherapy. 22Rv1 cells were treated daily with 1 μg/ml of phleomycin for 6 weeks (6WP) or left untreated (WT, AM). RNA was extracted and sequenced on the Illumina NovaSeq 6000 platform by the AGRF to generate 100 bp single end reads. RNA-seq reads for A) *OGDHL* and B) *ASNS* following repeated DNA damage. Gene expression analysis of C) *OGDHL* and D) *ASNS* via RT-qPCR 24 hours following a single dose of 2 Gy RT. The mean and SEM from three biological replicates are shown, p-value determined by students t-test. E) CBioPortal overall survival data for prostate cancer patients with altered *ASNS*.

ASNS and OGDHL were identified in more than one of our omics data sets as modulated by repeated phleomycin treatment, and therefore promising candidates for further investigation. According to the RNA-seq data, both genes increased expression in response to repeated DNA damage and repair (Figure 6a and b). Next, we determined their expression 24 hours following exposure to a single dose of RT. Interestingly, in this scenario *OGDHL* and *ASNS* expression were significantly decreased (Figure 6c and d; *P* = 0.03 and 0.03 respectively). We then sought to determine the potential clinical relevance of altered expression of these genes. *OGDHL* and *ASNS* alterations and their association with prostate cancer patient survival (a proxy of tumour aggression) were investigated in cBioPortal. While no association was found between *OGDHL* status and survival (data not shown) a significant association was found between *ASNS* status and patient survival (Figure 6e, *P* = 1.993e-4). It should be noted only 24 patients had an *ASNS* alteration (generally amplification, shown in supplementary Figure S3), versus 1451 in the unaltered group.

## Discussion

Understanding the underlying molecular characteristics of aggressive cancer cells remains one of the greatest obstacles in cancer treatment. Here we show that cancer cells surviving repeated DNA damage have altered molecular profiles along with increased resistance and metastatic potential. Our data is the first to profile an isogenic model of treatment resistance using RNA-seq and DNA methylation profiling. It has provided critical insight into the global response and adaptation cells exhibit to counter DNA damage.

Our findings clearly support the literature and the hypothesis that cells surviving repeated DNA damage display increased migration [1,2,8]. Currently, the effect of DNA damage on the epigenetic landscape is poorly understood. Epigenetics determines chromatin compaction and underlies the regulation of gene expression. Therefore, methylation changes following DNA damage have the potential to alter future treatment response and cancer progression and may represent novel candidate markers. As a single dose of irradiation has been shown to cause stable epigenetic changes [3], the cumulative impact of DNA damage on methylation was expected. Our data demonstrate that extended periods in cell culture increase DNA methylation and the addition of DNA damage increases DNA methylation further. Increased DNA methylation has been associated with more aggressive disease, supporting our findings [9]. A growing body of evidence indicates that DNA methylation can occur at sites of DSB repair [10,11], the increase in DNA methylation may therefore reflect *de novo* DNA methylation surrounding DSBs. Furthermore, DNA damage by phleomycin likely occurs at random sites as there were no convincing sets of CpGs shared between biological replicates. It should be noted that the Illumina EPIC 850k array covers a small percentage of all CpGs, meaning many potentially impacted CpGs were undetected. In future experiments, profiling the methylome of single cells would be beneficial as opposed to an average of the cell population. While cells were selected based on their ability to survive phleomycin treatment, the surviving cell population would still exhibit genetic and epigenetic heterogeneity.

While DNA damage sites may be random in response to chronic DNA damage, the majority of CpGs with altered methylation were in gene bodies rather than gene promoters. Gene bodies are often in regions of euchromatin and previous studies have shown that euchromatin is more susceptible to DSB induction than heterochromatin [12,13]. Therefore, if the corresponding genes were being actively transcribed and were in an open euchromatin state they may have been hotspots for DNA damage [3]. However, the Illumina EPIC platform is also biased towards gene body CpGs, with 35.9% of probes located in this functional region which could have impacted results [14]. While most evidence associates gene body hypermethylation with increased gene expression, precisely how gene body methylation contributes to gene regulation remains unclear [15]. Taken as a whole, the differentially methylated genes in the 6 week treated cells were predominantly hypermethylated. This supports previous studies which have described hypermethylation of radioresistant cell lines and tumours compared to radiosensitive samples [3,16,17].

In relation to the efficiency of DNA repair, none of the most differentially methylated CpGs between wild type and treated cells were associated with DNA repair genes. Likewise, gene ontology analysis did not identify enrichment in DNA repair pathways. These findings are in disagreement with Antwih and colleagues, where methylation changes were enriched in DNA repair pathways following DNA damage [18]. Instead, it is possible that methylation changes were completely random due to the random nature of DSB induction, rather than the consequence of a specific biological response to DNA damage.

The DNA methylation analysis following repeated DNA damage highlighted several altered CpGs including sites within the *OGDHL, FOXP1, RTN4,* and *EPHA4* genes. Two CpGs located in the *OGDHL* gene body displayed increased methylation after 6 weeks of DNA damage (cg08846770 and cg00898123 probes) and *OGDHL* was also identified as significantly differentially regulated via RNA-seq. OGDHL is a rate-limiting component of the oxoglutarate dehydrogenase (OGDH) complex which catalyzes the conversion of alpha-ketoglutarate to succinyl-CoA and CO_2_ during the TCA cycle [19,20].

The UPR was identified as the most significantly altered canonical pathway in our RNA-seq analysis. The UPR was also identified as enriched in the proteomics data set. Mounting evidence has demonstrated a critical role for the UPR in cancer progression, cell proliferation, survival, and treatment resistance [21,22]. Critically, the UPR also contributes to altered cell metabolism [21]. Briefly, the UPR is initiated upon accumulation of unfolded/misfolded proteins within the ER lumen. The UPR promotes cell survival in the presence of ER stress by reducing protein translation, upregulating chaperones and protein modifying enzymes, and enhancing the degradation of misfolded proteins [23]. Despite variable findings, altered expression of components of the UPR have been reported in numerous types of cancer and accumulating evidence suggests this may facilitate cancer development and progression [24–27]. Altered expression of components of the UPR may, therefore, serve as important biomarkers for treatment selection or may be targeted to overcome treatment resistance [23,28]. It is important to note that this can be a generalized stress response, so drilling down into the shared genes across multiple data sets is critical in identifying the most relevant candidates. Here we identified *ASNS* as central in the context of our model.

**Figure 7.**
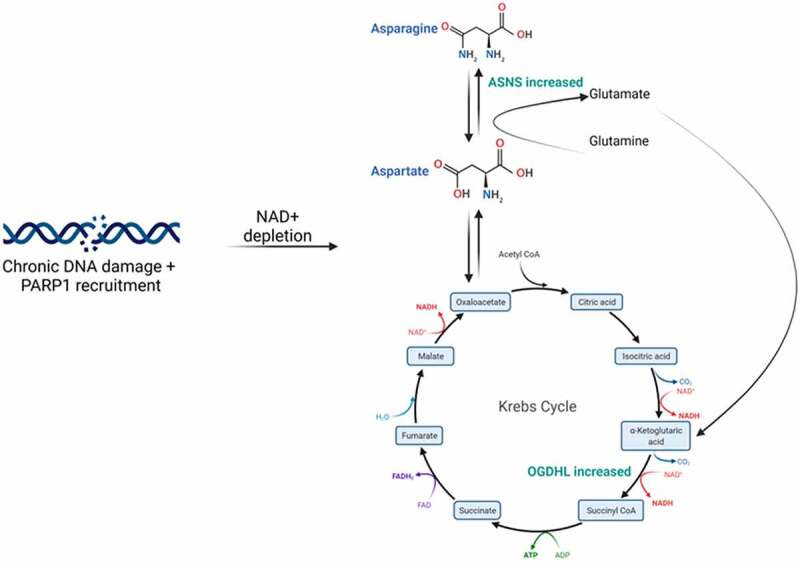
Proposed mechanism of chronic DNA damage on cell metabolism. Repeated DNA damage over time results in depletion of NAD+ due to the action of PARP1. The cell then compensates for this by attempting to increase TCA intermediates. The roles of ASNS and OGDHL are highlighted.

Our study highlighted *OGDHL* and *ASNS* as central candidates in an adaptive response to DNA damage and it is evident that an intricate link between DNA repair, the UPR, metabolism and epigenetics exists. The contrast between expression of *OGDHL* and *ASNS* in the repeated damage model versus radiotherapy experiments suggest that the initial or innate response of cancer cells to DNA damage is down-regulation of these genes. This is potentially followed by adaptive response to improve chances of cell survival. In response to DNA damage the UPR activates transcription of *ASNS* [29] and this increased *ASNS* activity can feed into the TCA cycle as glutamate (Figure 7). *OGDHL* may then respond to the increase in TCA cycle activity by increasing levels due to increased demand.

The precise link between this metabolic shift and surviving DNA damage needs to be determined, however, PARP1 likely plays a central role in this response. PARP1 is responsible for sensing DNA damage and was identified as significantly decreased in the proteomics data. TCA intermediates have been shown to inhibit PARP1 initiated cell death and hence contribute to a survival advantage in these cells [30]. Indeed, the answer may lie in work from Murata et al (2019). PARP1 is responsible for NAD+ depletion and has been shown to trigger a metabolic shift towards oxidative phosphorylation [31]. In cancer cells this initiates the Warburg reversing effect, however, the impact on treatment resistance needs to be investigated further. An increase in oxidative phosphorylation has previously been demonstrated in response to acute and chronic DNA damage and this was in response to the demand and decrease in NAD+ and ATP required by PARP-1 [32] (Figure 7). The unexpected decreased abundance of PARP1 24 hours post-RT detected in the proteomics data may be a compensatory mechanism induced by the cell to recover from the metabolically exhaustive process of repairing DNA damage. PARP1 consumes substantial amounts of NAD+ immediately following and during its activation, rapidly depleting the pool available for undertaking normal metabolic processes and endangering cell viability.

Integrating the RNA-seq and proteomics data sets, also provided some insight into the propensity for bone metastasis in prostate cancer. The epithelial to mesenchymal transition (EMT) marker vimentin (*VIM*) was found to be significantly *upregulated* in cells exposed to prolonged DNA damage. While VIM was also identified as significant in the proteomics data set, it exhibited *decreased* abundance post-RT and this fits with the patterns observed for *ASNS* and *OGDHL* between the two models mentioned previously. VIM is an intermediate filament ubiquitously expressed by mesenchymal cells [33]. High expression of VIM is often detected in poorly differentiated prostate cancers and bone metastases [34,35]. In contrast, VIM is nearly undetectable in well differentiated non-metastatic prostate tumours [34,35]. Methyltransferase-like protein 7A (*METTL7A*) and 7B were also identified in both data sets. *METTL7A* has recently been linked to osteogenic differentiation under metabolic stress and promotion of cell survival [36] along with methotrexate resistance [37]. This could indicate that DNA damage is associated with the initiation of metastasis to bone. Importantly, *METTL7B* can also be linked to our metabolic candidates as increased expression has been shown to increase glutathione metabolism-related process [38] with glutathione being composed of glutamate, the by-product of ASNS and precursor to the substrate of OGDHL, alpha-ketoglutarate.

Collectively, our data demonstrates that repeated DNA damage drives the evolution of cancer cells towards a more aggressive phenotype with increased DNA methylation evident along with altered gene expression. This indicates that cancer cells undergo epigenetic reprogramming during cancer treatment delivered via fractionated or repeated doses. This response may serve as a protective mechanism to allow cancer cells to survive treatment. The alteration of genes involved in the UPR and cell metabolism suggests clinical screening or dietary supplementation may benefit response to DNA damaging therapies. Future investigations into functional relevance will serve to clarify the true role of these dysregulated pathways.

## Methods

### Cell culture

22Rv1 cells, derived from a primary prostate tumour, were cultured in Roswell Park Memorial Institute medium 1640 (RPMI) (Sigma-Aldrich, USA). Medium was supplemented with 10% heat-inactivated foetal bovine serum (FBS) (Bovogen Biologicals, Australia) and 10 ml of 5000 U/ml penicillin and 5000 µg/ml streptomycin stabilized solution (Sigma-Aldrich, USA).

### Cell treatments

22Rv1 cells were treated daily with the radiomimetic phleomycin (Sapphire Biosciences, Australia). Cells were grown in 6-well plates (Corning Incorporated, USA) and treated with phleomycin for 1 hour at a final concentration of 1 µg/ml. Following treatment, medium containing phleomycin was aspirated, the cell layer was washed with PBS and fresh medium was added. Treatment was continued 24 hours apart for 6 weeks (week days only), to mimic the typical radiation therapy regimen experienced by prostate cancer patients. Age matched cells were grown in parallel to determine changes due to time in cell culture. Cells were harvested or treated for downstream assays immediately. Cell irradiations were carried out as described previously [4].

### Clonogenic assay

The clonogenic assay was performed as previously described [4] with 1000 cells plated into wells of a 6 well plate (each well with 35 mm diameter) and treated for 1 hour with phleomycin at 1, 10, 50, and 100 µg/ml and vehicle (PBS).

### Scratch assay

Cells were seeded onto Lab-Tek II 8-well chamber slides (Thermo Fisher Scientific, USA) and allowed to form a confluent monolayer. A 200 µl pipette tip was used to scratch the cell monolayer in each well. Cells were imaged at 10 × magnification using a Nikon Eclipse Ti-2 Inverted Microscope then incubated under standard conditions. Cell migration into the scratched area was documented over 48 hours. Images were analysed using the measuring tools in Fiji Image J. Cell migration at 48 hours was calculated by subtracting the width of the scratch at 48 hours from the width of the scratch at 0 hours. Cell migration was then divided by the width of the scratch at 0 hours to determine the percentage closure.

### Immunofluorescence staining

Cells were seeded onto Nunc® Lab-Tek® II Chamber Slides™ (Thermo Fisher Scientific, USA) and grown to 70% confluency. Cells were either left untreated or treated with phleomycin (1 µg/ml) for 1 hour. Medium was removed and cells were washed in PBS. Cells were then fixed in 4% formaldehyde (Cell Signaling Technology, USA) for 10 minutes. Fixed cells were washed twice for 5 minutes in PBS. Permeabilisation buffer containing 0.1% Triton-X (Sigma-Aldrich, USA) in PBS was added to each well and incubated for 10 minutes on a rotator at low speed. Permeablisation buffer was removed and cells were washed twice in PBS. Blocking buffer containing 0.1% Tween-20 (Sigma-Aldrich, USA) and 5% FBS was added and incubated at room temperature for 1 hour. Blocking buffer was removed and primary antibody was added and incubated overnight at 4°C. The next day, all wells were washed 3 times in blocking buffer and secondary antibody was added to each well. The chamber slide was wrapped in foil and incubated at room temperature for 1 hour. Wells were then washed 3 times in PBS and DAPI/Fluroshield (Abcam, UK) were added to the slide prior to coverslipping. Slides were imaged using a Nikon Eclipse Ti-2 Inverted Microscope at 40 × magnification with an exposure time of 100 ms.

### DNA methylation analysis – cell lines

DNA was extracted using the DNeasy Blood & Tissue Kit (50) (Qiagen, USA) according to the manufacturer’s instructions. DNA was eluted using 200 μl of elution buffer and was quantified using a NanoDrop® ND-1000 spectrophotometer (NanoDrop® Technologies, USA). DNA was sent to the Australian Genome Research Facility (AGRF) and run on the Illumina Infinium MethylationEPIC BeadChip (Illumina 850k array) (Illumina, USA) platform. All analyses were undertaken using the R statistical environment (v3.5.0) and QC and probe summaries were assessed using the ‘lumi’ Bioconductor package [39]. The detection P-values for each sample were plotted to identify any failed samples and Subset-quantile Within Array Normalisation (SWAN) was applied to raw data. Probes that had failed in one or more samples were removed from the analysis. Probes with SNPs at the CpG site, those associated with the sex chromosomes, and those shown to be cross-reactive were also removed [40]. M-values and beta-values were calculated for 815,228 probes and heat maps of the top 50 most differentially methylated CpG sites between WT and 6WP groups were generated using R.

### DNA methylation analysis – patient samples

Data analysis for the OGDHL probe was run on previously published data from Wilkinson et al [7]. Briefly, archived pathology blocks were obtained from pathology laboratories Tasmania, Human Research Ethics Committee (Tasmania) (ethics number H9999, H0017040). DNA was extracted from Formalin fixed paraffin embedded matched localized prostate tumour and bone metastasis pathology specimens (eight samples total). Sections (5 μm) were dewaxed in Xylene, incubated at 56^o^ C in Proteinase K for 24 hours. DNA was extracted using the QIAamp DNA FFPE tissue kit (QIAGEN) according to the manufacturer’s instructions. DNA (500 ng) was bisulphite converted using the EZ DNA Methylation-Gold Kit according to the manufacturer’s instructions (Zymo research, USA). DNA methylation was then profiled using the Illumina Methylation EPIC array (Illumina, CA, USA) at the AGRF. Data was analysed using Illumina’s GenomeStudio v2011.1 with Methylation module 1.9.0 software and illumina MethylationEPIC_v-1-0_B3 manifest file. Raw intensity data (IDAT) files were imported into R studio (version 3.4.0) using the minfi package (version 1.24.0) [41] and processed using missMethyl Bioconductor package (version 1.12.0) [42]. Data were processed with subset quantile within array normalization (pre-processSWAN) [43].

### RNA-seq

RNA was extracted using TRI reagent®. RNA samples underwent clean-up using the RNeasy Plus Micro Kit (50) (Qiagen, USA) following the manufacturer’s instructions. Samples were prepared for sequencing by the AGRF and 100 bp single end reads were obtained using the Illumina NovaSeq 6000 platform (Illumina, San Diego, USA). Image analysis was conducted in real time by the NovaSeq Control Software (v1.6.0) and Real Time Analysis (v3.4.4) which performs base calling. The Illumina bcl2fastq 2.20.0.422 pipeline was used to generate sequence data.

Data processing and bioinformatics analysis were also performed by the AGRF. The RNA-seq expression analysis workflow included alignment against the human reference genome version hg38 using STAR aligner (v2.5.3a). This was followed by transcript assembly using stringtie tool (v1.3.3) then quantification and Trimmed Mean of M-values (TMM) normalization. Differential expression analysis was performed using edgeR (v3.22.3) and R (v3.5.0).

### Ingenuity Pathway Analysis (IPA)

The Ingenuity Pathway Analysis program (QIAGEN Inc., https://www.qiagenbioinformatics.com/products/ingenuitypathway-analysis) was used to perform a core analysis on the gene files generated by the AGRF. Gene ID, log2 fold change (>1) and adjusted p-value (<0.05) for the top 500 differentially expressed genes (WT vs 6WP) were uploaded to IPA. Direct and indirect relationships between significant genes were considered when performing the analysis.

### Proteomics profiling

Protein was extracted from untreated and irradiated (2 Gy) 22Rv1 cells by lysis in denaturation buffer composed of 7 M urea, 2 M thiourea and 40 mM Tris (pH 7.5) including protease inhibitors (cOmplete Mini ETDA-free cocktail, Merck). Following protein quantitation using the A660nm assay (Pierce), 30 μg aliquots of protein were sequentially reduced and alkylated using standard protocols, then digested with 1.2 μg proteomics grade trypsin/rLysC (Promega) using the SP3 method (Hughes et al). Digests were desalted using C18 ZipTips (Merck) according to manufacturer’s instructions dried in a SpeedVac then reconstituted in 12 μl HPLC loading buffer, then Peptide samples were analysed by nanoflow HPLC-MS/MS using an Ultimate 3000 nano RSLC system (Thermo Fisher Scientific) coupled with a Q-Exactive HF mass spectrometer fitted with a nanospray Flex ion source (Thermo Fisher Scientific, Waltham, USA) and controlled using Xcalibur software (version 4.3). Approximately 1 mg of each sample was injected and separated using a 120-min segmented gradient. Peptides were preconcentrated onto a 20 mm × 75 µm PepMap 100 C18 trapping column then separated on a 250 mm × 75 µm PepMap 100 C18 analytical column at a flow rate of 300 nL/min and held at 45°C. MS Tune software (version 2.9) parameters used for data acquisition were: 2.0 kV spray voltage, S-lens RF level of 60 and heated capillary set to 250°C. MS1 spectra (390–1240 m/z) were acquired at a scan resolution of 120,000 in profile mode with an AGC target of 3e6 and followed by sequential MS2 scans across 26 DIA × 25 amu windows over the range of 397.5–1027.5 m/z, with 1 amu overlap between sequential windows. MS2 spectra were acquired in centroid mode at a resolution of 30,000 using an AGC target of 1e6, maximum IT of 55 ms and normalized collision energy of 27. DIA-MS data were generated for three biological replicates of each cell type. Data are available via ProteomeXchange with identifier P × D033400.

DIA-MS raw files were processed using Spectronaut software (version 15.0) (Biognosys AB, Wagistrasse, SWI). A project-specific library was generated using the Pulsar search engine to search the DIA MS2 spectra against the Homo sapiens UniProt reference proteome comprising 20,443 entries (downloaded December 2019). With the exception that single-hit proteins were excluded, default (BGS factory) settings were used for both spectral library generation and DIA data extraction. For library generation, N-terminal acetylation and methionine oxidation were included as variable modifications, cysteine carbamidomethylation was specified as a fixed modification and up to two missed cleavages were allowed. Peptide, protein and PSM thresholds were set to 0.01. Mass tolerances were based on first-pass calibration and extensive calibration for the calibration and main searches, respectively, with correction factors set to 1 at the MS1 and MS2 levels. Targeted searching of the library based on XIC extraction deployed dynamic retention time alignment with a correction factor of 1. Protein identification deployed 1% q-value thresholds at the precursor and protein levels, respectively, and automatic generation of mutated peptide decoys based on 10% of the library and dynamic decoy limitation for protein identification. Relative peptide quantitation between experimental samples deployed the MaxLFQ algorithm, using the intensity values for the Top3 peptides (stripped sequences) and cross-run normalization based on median peptide intensity.

Spectronaut reports were imported into Perseus software (Tyanova et al., 2016) for statistical analysis. Missing values, which comprised of <1% of the total protein number, were imputed using default Perseus settings. Proteins with significant differences in abundance across the two sample groups were determined by t-test analysis (FDR <0.05; s0 < 0.1).

### RT-qPCR analysis

RNA was converted to cDNA using the SuperScript™ III First-Strand Synthesis System. cDNA was amplified on the Rotor-Gene 2000 real-time cycler (Corbett Research, Australia) using Quantitect SYBR Green PCR mastermix (Qiagen, Germany). Primers sequences are in Table 2. Cycling conditions were 95°C for 15 minutes followed by 40 cycles of 95°C for 15 seconds then 60°C for 60 seconds. Melt analysis was used to confirm the presence of a single PCR product.

**Table 2.** Primer sequences for RT-qPCR.

Primer	Sequence (5’ to 3’)
ASNS Forward	CGACCAAAAGAAGCCTTCAG
ASNS Reverse	CTGGGTAATGGCGTTCAAAG
OGDHL Forward	CCCTGACCTGCAGCCTCC
OGDHL Reverse	CCTCAGCTGACTCATTCGGG
GAPDH Forward	AAATATGATGACATCAAGAAGGTGGT
GAPDH Reverse	AGCCCAGGATGCCCTTGAGGG

### Statistical analysis

Graphs were generated and statistical analyses performed using GraphPad Prism version 8.2 for Mac OSX, GraphPad Software, La Jolla California USA. Clonogenic data was analysed by two-way ANOVA and Sidak’s multiple comparisons test. A Student’s one-tailed t-test was used for analysis of scratch assay data. For real-time PCR, a Student’s t-test was used. A p-value ≤0.05 was considered significant for these analyses.

## Supplementary Material

Supplemental Material

## Data Access statement

RNA-seq data is available at GSE208190 and DNA methylation data is available at GSE208189. Proteomics data is available via ProteomeXchange with identifier P × D033400.
