# Supplementary material for: Multiomics analysis of adaptation to repeated DNA damage in prostate cancer cells
Source: Epigenetics. 2023 May 17;18(1):2214047. doi: 10.1080/15592294.2023.2214047 (PMC10193866; doi:10.1080/15592294.2023.2214047)
Supplement: Supplemental Material [file KEPI_A_2214047_SM5927.zip › Supplementary files/Supplementary Table and Figure Legends.docx]

**Supplementary Table and Figure Legends**

**Supplementary Figure 1. Phleomycin treatment induces DSBs.**

Gamma H2A.X immunofluorescence staining was performed on 22Rv1 cells either left untreated or treated with 1ug/ml phleomycin. Green = gamma H2A.X Blue= DAPI.

**Supplementary Table 1.** Gene names for the top 50 differentially methylated CpGs (note some genes were not identifiable/listed NA in heatmap).

**Supplementary Figure 2. The top 50 DNA methylation changes which occur in cell culture in the absence of DNA damage (age matched cells).**

22Rv1 cells were treated daily with 1 μg/ml of phleomycin for 6 weeks (6WP) or left untreated (WT, AM). DNA was extracted and DNA methylation was profiled by the Australian Genome Research Facility (AGRF) using the Illumina EPIC 850k array. Heatmap depicts top 50 most differentially methylated CpGs. Red indicates high levels of methylation and blue low methylation relative to the mean for a given CpG. CpGs not associated with a known gene are labelled ‘NA’

**Supplementary Figure 3. *ASNS* alterations identified from CBioPortal data.**

CBioPortal data for prostate cancer patients with altered *ASNS*. A) Studies selected for analysis. B) Screen capture of *ASNS* alterations across the 9041 patients. From the 1.8% with alterations in *ASNS*, the majority exhibited gene amplification.
