# Supplementary figures and images for: Multiomics analysis of adaptation to repeated DNA damage in prostate cancer cells
Source: Epigenetics. 2023 May 17;18(1):2214047. doi: 10.1080/15592294.2023.2214047 (PMC10193866; doi:10.1080/15592294.2023.2214047)

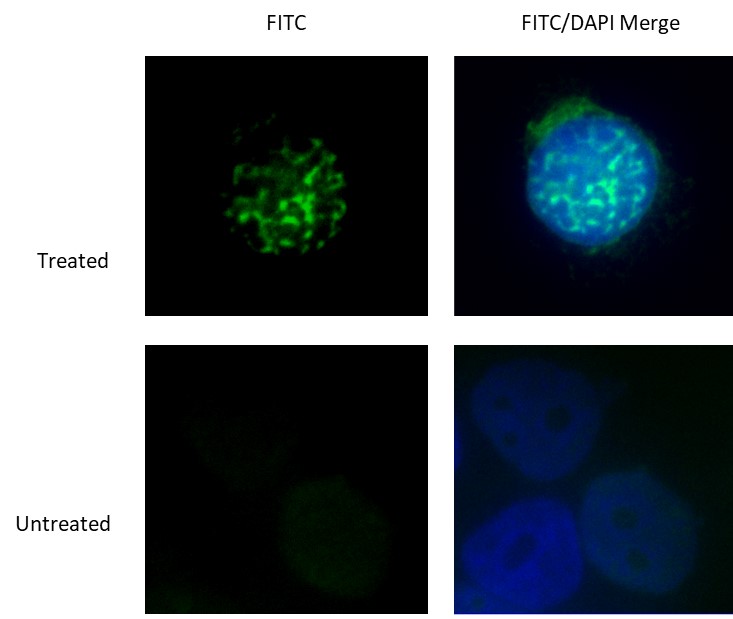

Supplement: Supplemental Material [file KEPI_A_2214047_SM5927.zip › Supplementary files/Figure S1.jpg]

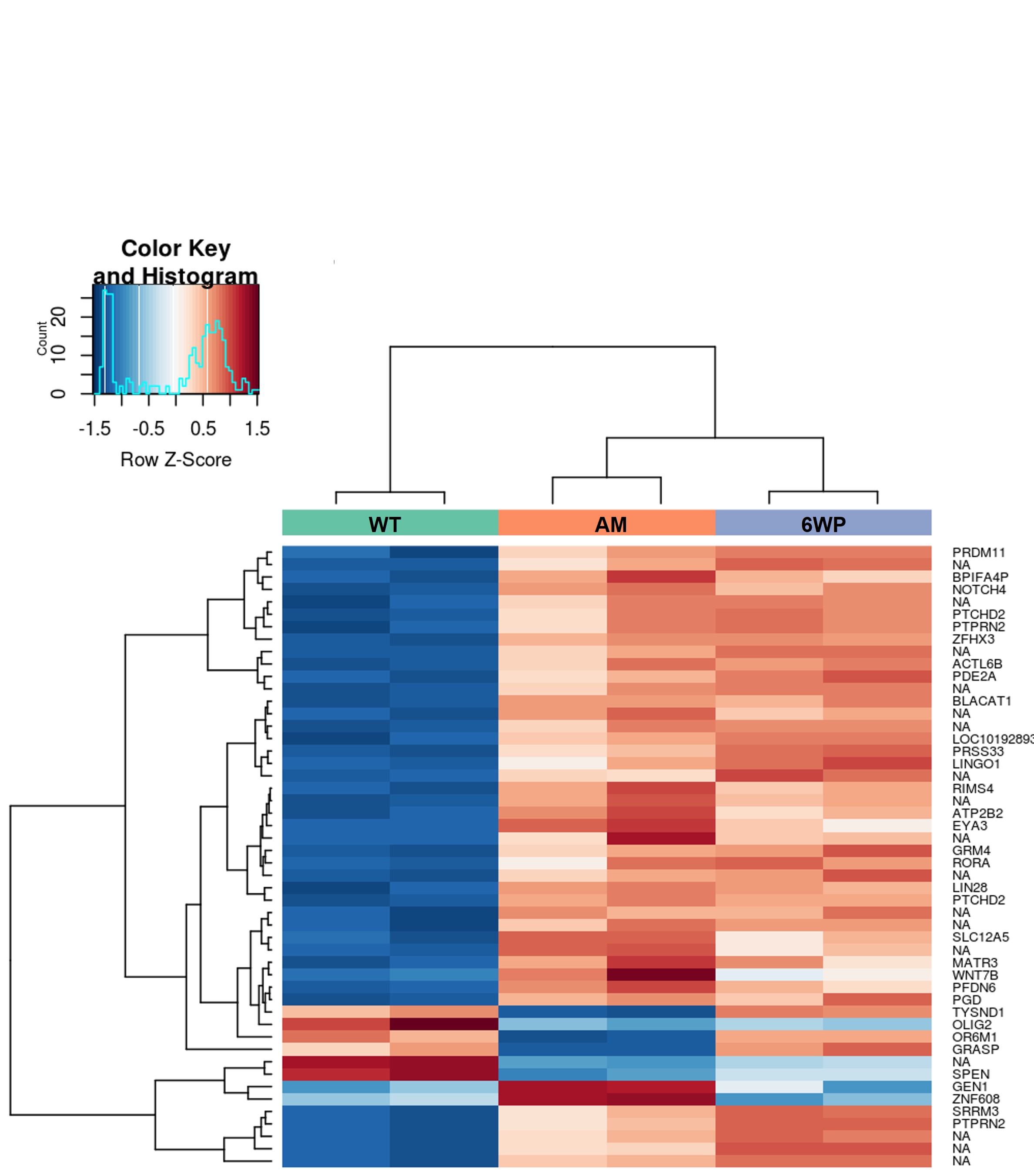

Supplement: Supplemental Material [file KEPI_A_2214047_SM5927.zip › Supplementary files/Figure S2.jpg]

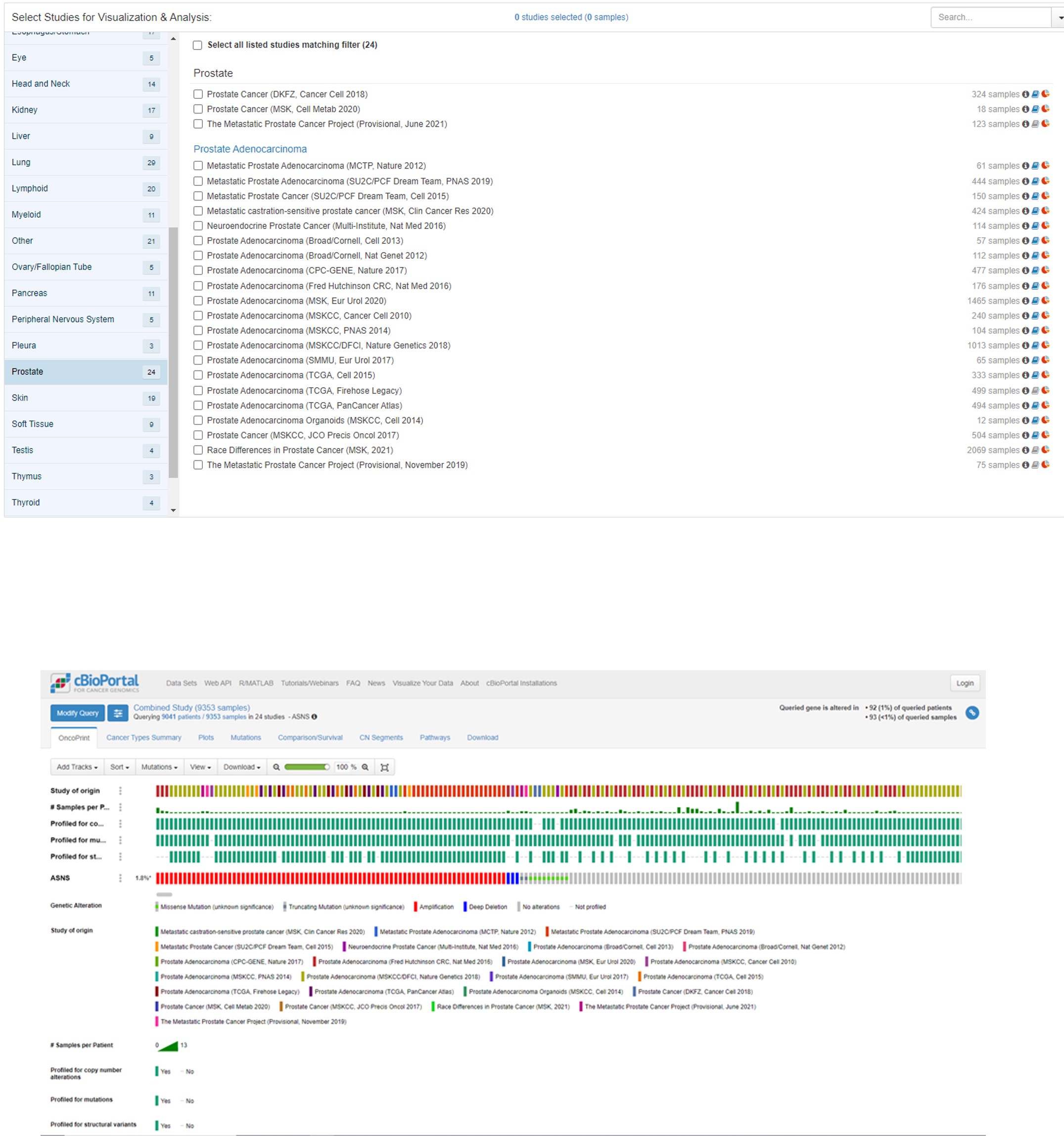

Supplement: Supplemental Material [file KEPI_A_2214047_SM5927.zip › Supplementary files/Figure S3.jpg]
